# Spatiotemporal dynamics of the CD11c^+^ microglial population in the mouse brain and spinal cord from developmental to adult stages

**DOI:** 10.1186/s13041-024-01098-2

**Published:** 2024-05-18

**Authors:** Kohei Nomaki, Risako Fujikawa, Takahiro Masuda, Makoto Tsuda

**Affiliations:** 1 Department of Molecular and System Pharmacology, Graduate School of Pharmaceutical Sciences, Kyushu University, 3-1-1 Maidashi, Higashi-ku, Fukuoka, 812-8582 Japan; 2 Division of Molecular Neuroimmunology, Medical Institute of Bioregulation, Kyushu University, 3-1-1 Maidashi, Higashi-ku, Fukuoka, 812-8582 Japan; 3 Kyushu University Institute for Advanced Study, 744 Motooka Nishi-ku, Fukuoka, 819-0395 Japan

**Keywords:** CD11c^+^ microglia, Spatiotemporal dynamics, Brain, Spinal cord, Pre/postnatal development, Adult, Mouse

## Abstract

CD11c-positive (CD11c^+^) microglia have attracted considerable attention because of their potential implications in central nervous system (CNS) development, homeostasis, and disease. However, the spatiotemporal dynamics of the proportion of CD11c^+^ microglia in individual CNS regions are poorly understood. Here, we investigated the proportion of CD11c^+^ microglia in six CNS regions (forebrain, olfactory bulb, diencephalon/midbrain, cerebellum, pons/medulla, and spinal cord) from the developmental to adult stages by flow cytometry and immunohistochemical analyses using a CD11c reporter transgenic mouse line, *Itgax-Venus*. We found that the proportion of CD11c^+^ microglia in total microglia varied between CNS regions during postnatal development. Specifically, the proportion was high in the olfactory bulb and cerebellum at postnatal day P(4) and P7, respectively, and approximately half of the total microglia were CD11c^+^. The proportion declined sharply in all regions to P14, and the low percentage persisted over P56. In the spinal cord, the proportion of CD11c^+^ microglia was also high at P4 and declined to P14, but increased again at P21 and thereafter. Interestingly, the distribution pattern of CD11c^+^ microglia in the spinal cord markedly changed from gray matter at P4 to white matter at P21. Collectively, our findings reveal the differences in the spatiotemporal dynamics of the proportion of CD11c^+^ microglia among CNS regions from early development to adult stages in normal mice. These findings improve our understanding of the nature of microglial heterogeneity and its dynamics in the CNS.

## Introduction

An increasing body of evidence indicates that microglia, the resident immune cells of the central nervous system (CNS), have diverse functions that critically contribute to CNS development, homeostasis, and disease [1, 2]. These include removal of apoptotic cells, survival of neuronal progenitors, axonal growth, synapse regulation, inflammatory responses, and neural damage/repair [1, 2]. Remarkable progress in our understanding of microglia has been made by the comprehensive characterization of individual microglia in rodents and humans [3–5] and their temporal changes during CNS development and disease [5–7], providing evidence that microglia exist in heterogeneous cell populations and states in these contexts [2, 8]. A notable example is disease-associated microglia (DAM), a population that appears in the brain of mouse models of Alzheimer’s disease (AD) [3]. One of the highly upregulated genes in DAM and similar microglial states is *Itgax* [3, 9–11], which encodes integrin αX (also known as CD11c). CD11c^+^ microglia [8, 11–15] and various other microglial populations (for example, axon tract-associated microglia (ATM) [7], proliferative region-associated microglia (PAM) [6], youth-associated microglia (YAM) [16], and Arginase-1^+^ microglia [17]) have also been found in the pre/postnatal developing and adult brains of mice and humans. The core signature gene shared by these populations is *Itgax* [6, 7, 11, 16, 17]. The role of each distinct microglial population in health and disease remains to be fully understood; however, CD11c^+^ microglia and similar populations have recently been implicated in pre/postnatal brain development [15, 17], myelination [12, 18], and CNS diseases [11] such as AD [3, 9, 14, 19], multiple sclerosis [9, 12, 18, 20], and chronic pain [21, 22].

Despite recent accumulating evidence for their potential as critical elements in CNS development, homeostasis, and disease, the dynamics of CD11c^+^ microglia in the brain and spinal cord during development and CNS diseases are not fully understood. It has previously been shown that the abundance of the CD11c^+^ population increases during early postnatal development and with aging [12, 13, 23]; however, the spatiotemporal dynamics of CD11c^+^ microglia in individual CNS regions remain to be determined. This seems to be important because recent studies have shown that the distribution of microglia is not uniform in each brain region during development, and that the uneven spatial pattern is implicated in site-specific structural maintenance and neuronal generation/migration/wiring [8, 15, 24].

Therefore, in this study, we investigated the proportion of CD11c^+^ microglia in six CNS regions (forebrain, olfactory bulb, diencephalon/midbrain, cerebellum, pons/medulla, and spinal cord) from the developmental to adult stages by flow cytometry and immunohistochemical analyses using a CD11c reporter transgenic mouse line, *Itgax-Venus*.

## Results

**Fig. 1:**
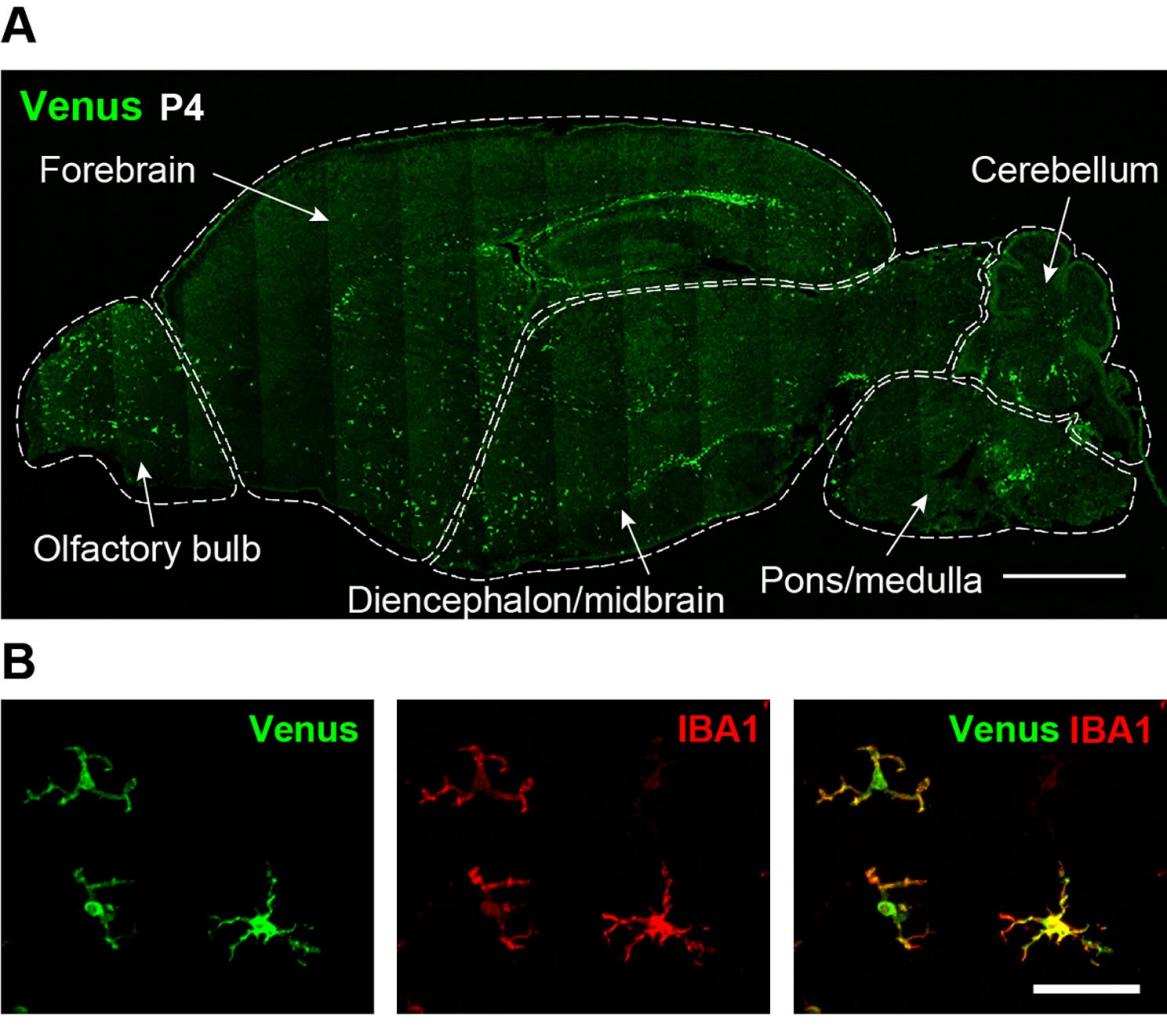
CD11c^+^ cells in the brain of *Itgax-Venus* mice at P4. (**A**) Representative immunofluorescence images of Venus^+^ (CD11c^+^) cells in the whole brain of *Itgax-Venus* mice at P4. Each brain region for quantitative analyses in further experiments is indicated by dashed lines. (**B**) Immunolabeling of Venus^+^ cells (green) with IBA1 (red) in the forebrain. Scale bars, 1000 μm (**A**) and 50 μm (**B**)

**Fig. 2:**
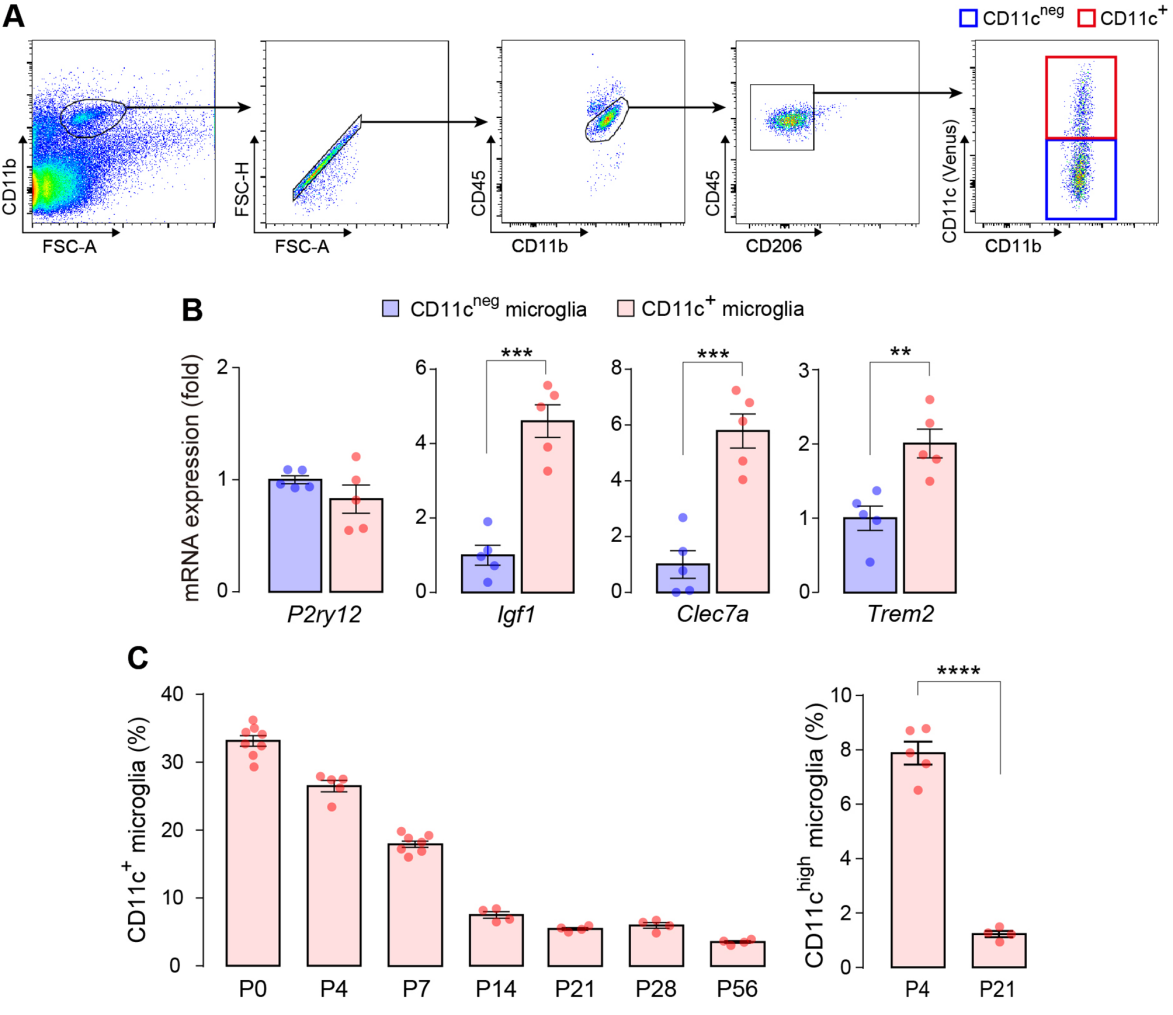
Flow cytometry analysis of the proportion of CD11c^+^ microglia in the forebrain. (**A**) Gating strategy for the CD11c^+^ and CD11c^neg^ microglia (CD11b^+^ CD45^low^ CD206^neg^ cells with and without Venus fluorescence in red and blue squares, respectively) in the forebrain of *Itgax-Venus* mice at P4. (**B**) qPCR analysis of *P2ry12*, *Igf1*, *Clec7a*, and *Trem2* mRNA expression in FACS-isolated CD11c^neg^ and CD11c^+^ microglia from the forebrain at P4 (*n* = 5 mice). Values represent the relative ratio of the mRNA levels (normalized to *Actb* mRNA) of the CD11c^neg^ microglia group. (**C**) Temporal analysis of the proportion of CD11c^+^ microglia in the forebrain from P0 to P56 (*n* = 4–8 mice for each time point tested). The proportion of CD11c^+^ microglia is indicated as the percentage of Venus^+^ cells in the total microglia (CD11b^+^ CD45^low^ CD206^neg^ cells). The proportion of CD11c^high^ microglia (the upper half of CD11c^+^ microglia in the scattered plot) to CD11c^+^ microglia at P4 and P21 was also analyzed. Data are shown as means ± SEM. **P < 0.01, ***P < 0.001, and ****P < 0.0001

**Fig. 3:**
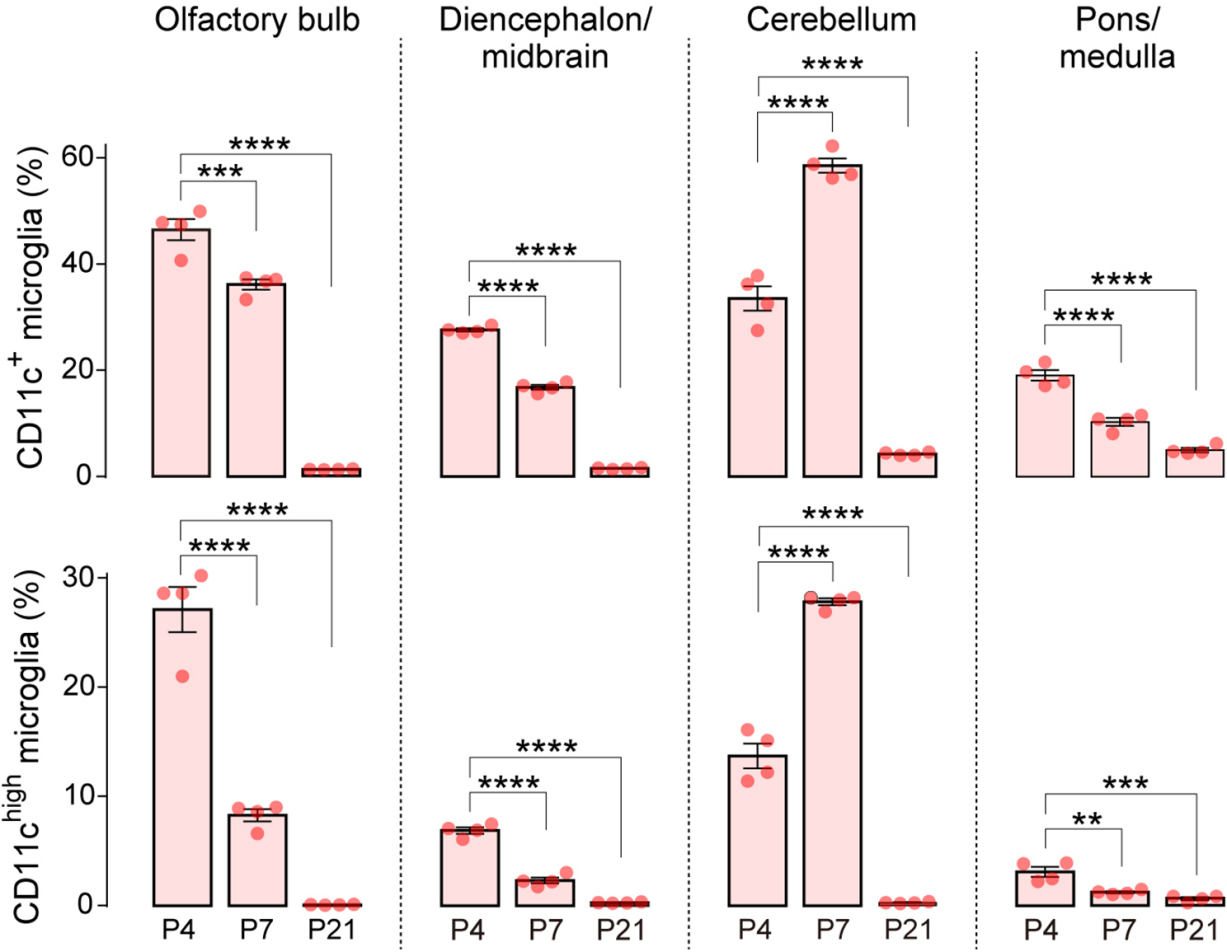
Spatiotemporal analysis of CD11c^+^ microglia in other brain regions. Flow cytometric analysis of the percentage of CD11c^+^ (upper panels) and CD11c^high^ (lower panels) microglia per total microglia (CD11b^+^ CD45^low^ CD206^neg^ cells) at P4, P7, and P21 (*n* = 4 mice at each time point). Data are shown as means ± SEM. **P < 0.01, ***P < 0.001, and ****P < 0.0001

To investigate the proportional dynamics of CD11c^+^ cells in the CNS, we utilized *Itgax-Venus* mice to enable the efficient detection of CD11c^+^ cells via expression of the fluorescent protein Venus, a reliable tool for studying CD11c^+^ microglia [21]. We first confirmed that Venus^+^ cells were present throughout the brain at postnatal day (P)4 (Fig. 1A) and almost all Venus^+^ cells were immunolabeled with ionized calcium-binding adapter molecule 1 (IBA1), a widely known microglial marker, which is consistent with previous data [12, 18] (Fig. 1B). As Venus^+^ cells were unevenly distributed in the brain at P4, we further examined the proportion of Venus^+^ cells spatially and temporally after birth in five individual regions (forebrain, olfactory bulb, diencephalon/midbrain, cerebellum, and pons/medulla) by quantitatively analyzing the percentage of Venus^+^ cells in each region using flow cytometry. Based on previous data indicating the presence of non-microglial CD11c^+^ macrophages in the choroid plexus and meninges [25], we analyzed the CD11b^+^ CD45^low^ CD206^neg^ Venus^+^ singlet population (Fig. 2A), which corresponds to CD11c^+^ microglia. CD11c^+^ microglia were detected in all the brain regions described above. In the forebrain at P4, we confirmed that the CD11c^+^ and CD11c^neg^ microglia collected by fluorescence-activated cell sorting (FACS) expressed *P2ry12* mRNA (a microglial gene). Compared to CD11c^neg^ microglia, CD11c^+^ microglia highly expressed the mRNAs of *Igf1*, *Clec7a*, and *Trem2* (characteristic genes expressed in CD11c^+^ microglia under normal and disease conditions [3, 11, 12]) (Fig. 2B). The proportion of CD11c^+^ microglia to total microglia was approximately 30% at P0/P4. After P4, the CD11c^+^ proportion decreased to < 10% by P14, and this low level persisted beyond P56 (the last time point tested) (Fig. 2C). In addition, the percentage of the CD11c^high^ population (the upper half of CD11c^+^ microglia in the scattered plot) to total CD11c^+^ microglia was also much lower at P21 than at P4, suggesting that the entire CD11c^+^ population changed toward CD11c^neg^ (Fig. 2C). CD11c^+^ cells were found in all other brain regions (olfactory bulb, diencephalon/midbrain, cerebellum, and pons/medulla) at P4, but the proportion of CD11c^+^ microglia varied (Fig. 3). At P4, the highest proportion was observed in the olfactory bulb, where half of the microglia were CD11c^+^. The temporal pattern of the CD11c^+^ proportion was similar (but slightly different) in most regions. However, in the cerebellum, a proportional peak was observed at P7. The temporal change in the CD11c^high^ proportion in all brain regions was similar to that of the CD11c^+^ proportion (Fig. 3). These data suggest that the abundance ratio and temporal dynamics of CD11c^+^ microglia are not uniform among these brain regions during postnatal development.

**Fig. 4:**
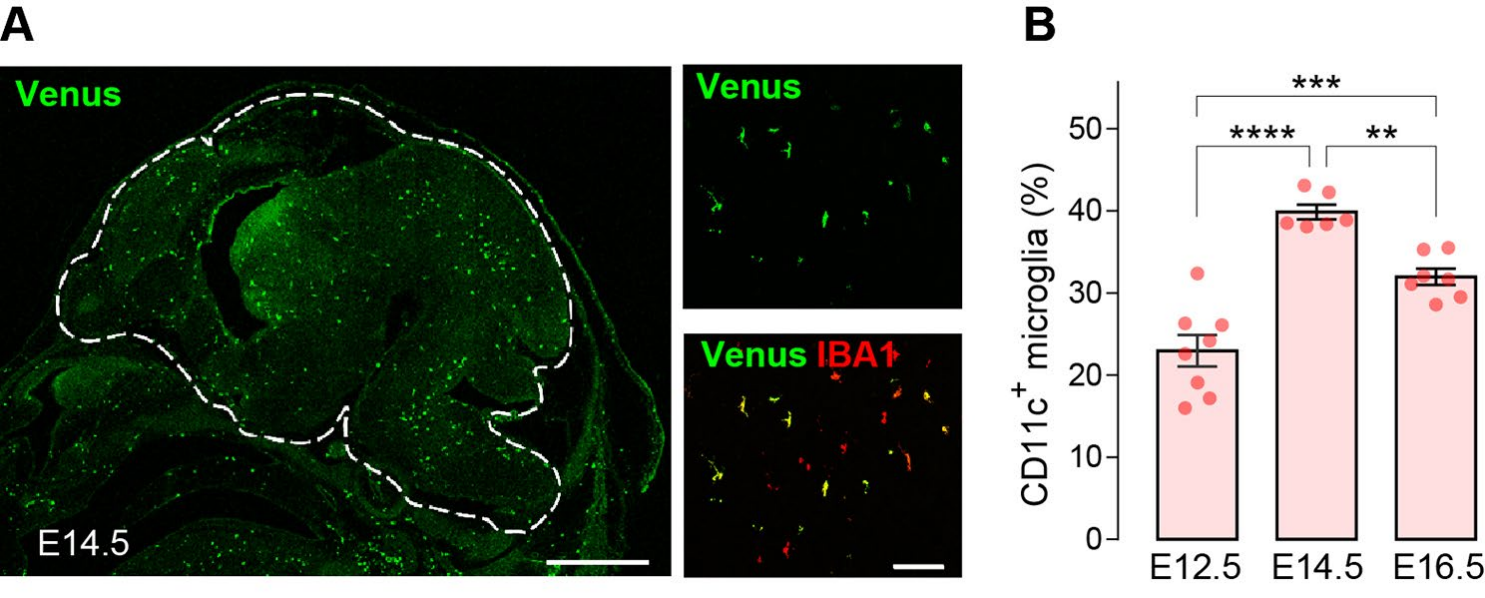
CD11c^+^ microglia in the prenatal mouse brain. (**A**) Representative immunofluorescence images of Venus^+^ (CD11c^+^; green) cells in the whole brain of *Itgax-Venus* mice at E14.5. The parenchymal brain region was indicated by a dashed line. Venus^+^ cells (green) were colocalized with IBA1 (red). Scale bars, 1000 μm (left panel) and 100 μm (right panel). (**B**) Flow cytometry analysis of the percentage of CD11c^+^ microglia per total microglia (CD11b^+^ CD45^low^ CD206^neg^ cells) at E12.5, E14.5, and E16.5 (*n* = 6–8 mice for each time point tested). Data are shown as means ± SEM. **P < 0.01, ***P < 0.001, and ****P < 0.0001

Our data obtained from the forebrain, showing that the proportion of CD11c^+^ microglia was the highest at P0, raises the possibility that CD11c^+^ microglia are proportionally abundant during prenatal stages. CD11c^+^ (Venus^+^) cells with IBA1 expression were found in all brain regions with an uneven distribution at embryonic day (E)14.5 (Fig. 4A). We did not analyze these cells in individual regions by flow cytometry because of the difficulty in precisely dividing each region of the embryonic brain. We found, however, that CD11c^+^ microglia were already present in the whole brain at E12.5, and that their proportion increased at E14.5 and slightly decreased at E16.5 (Fig. 4B). These data suggest that one-third of the total microglia in the entire brain have already acquired the CD11c^+^ state at E14.5, and that this state spans the pre- and postnatal stages.

**Fig. 5:**
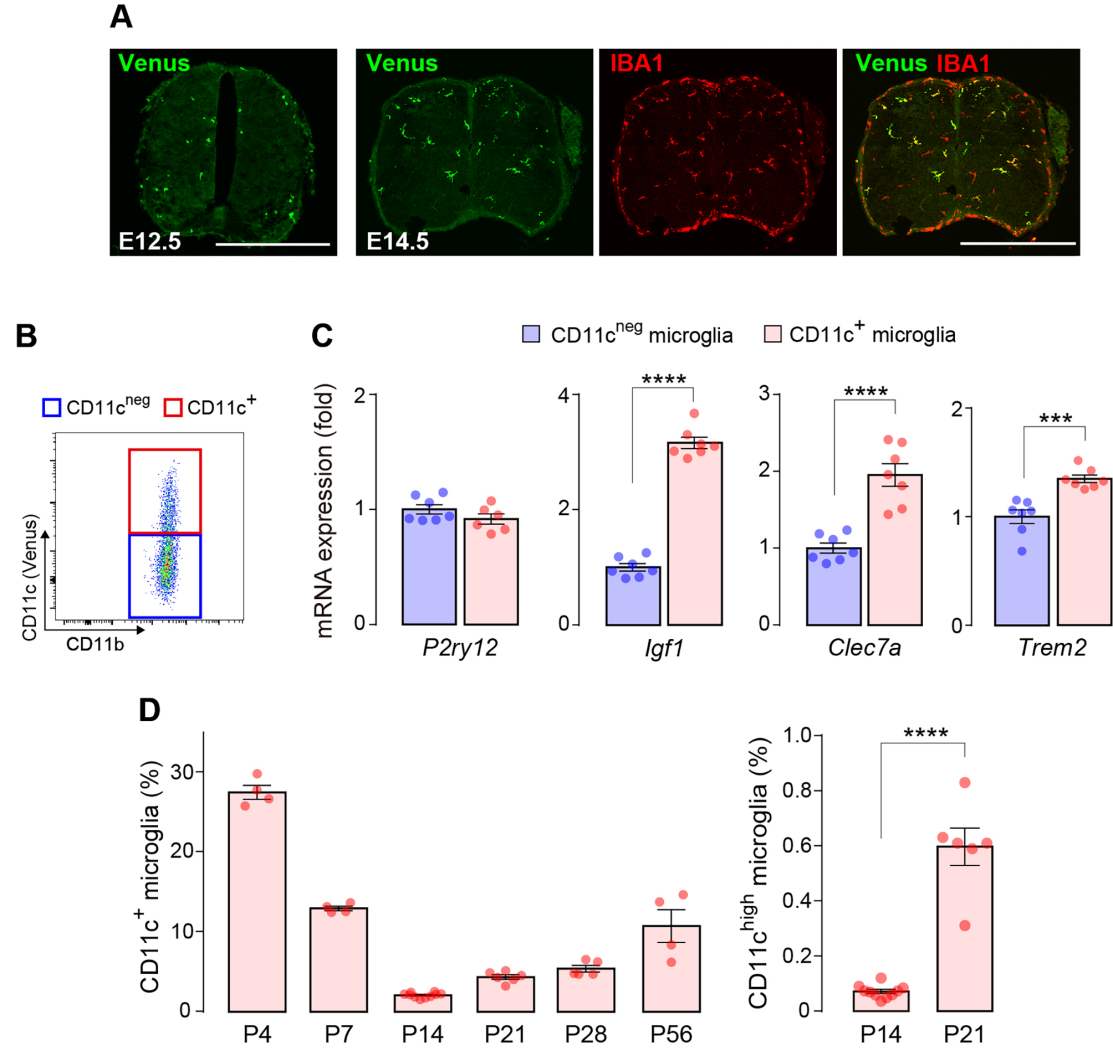
Temporal analysis of CD11c^+^ microglia in the mouse spinal cord. (**A**) Representative immunofluorescence images of Venus^+^ (CD11c^+^; green) cells in the spinal cord of *Itgax-Venus* mice at E12.5 and E14.5. Venus^+^ cells (green) were colocalized with IBA1 (red) in the spinal cord. Scale bars, 500 μm. (**B**) Representative scattered plot of CD11c^neg^ and CD11c^+^ microglia (blue and red square, respectively) in the spinal cord of *Itgax-Venus* mice at P4. (**C**) qPCR analysis of *P2ry12*, *Igf1*, *Clec7a*, and *Trem2* mRNA in FACS-isolated CD11c^neg^ and CD11c^+^ microglia in the spinal cord at P4 (*n* = 7 mice). Values represent the relative ratio of the mRNA levels (normalized to *Actb* mRNA) of CD11c^neg^ microglia group. (**D**) Temporal analysis of the percentage of CD11c^+^ and CD11c^high^ microglia per total microglia (CD11b^+^ CD45^low^ CD206^neg^ cells) during development and adult (*n* = 4–10 mice for each time point tested). Data are shown as means ± SEM. ***P < 0.001, and ****P < 0.0001

**Fig. 6:**
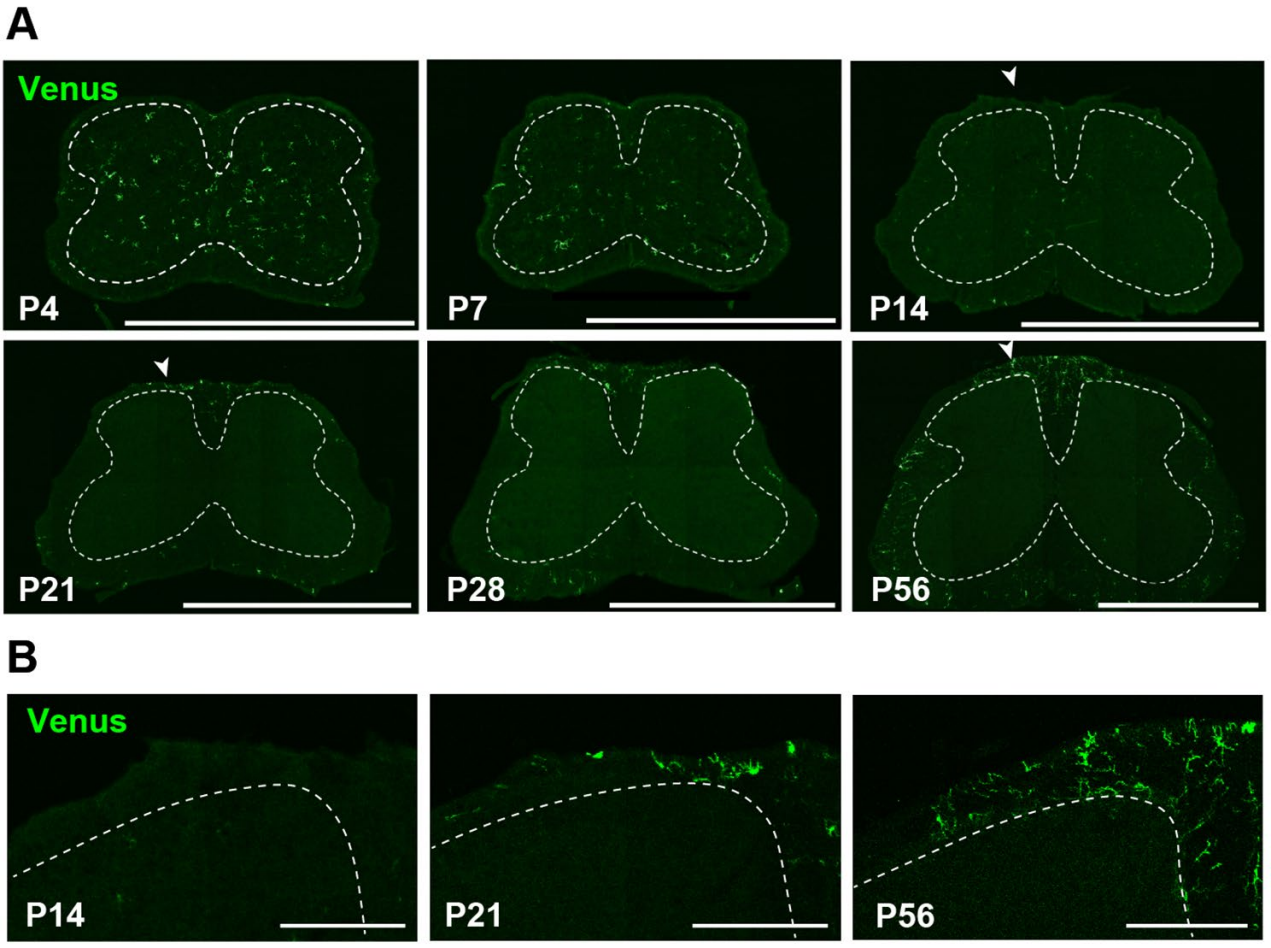
Spatial changes in spinal CD11c^+^ microglia during development. (**A**) Representative immunofluorescence images of Venus^+^ (CD11c^+^; green) cells in the spinal cord of *Itgax-Venus* mice at P4, P14, P7, P21, P28, and P56. GM regions are indicated by dashed lines. (**B**) High magnification images of the areas (indicated by white arrowheads in panel **A**). Dashed lines indicate the boundary between WM and GM. Scale bars, 1000 μm (**A**) and 200 μm (**B**)

To further characterize the CD11c^+^ microglia dynamics in the CNS, we analyzed the spinal cord, a region that has not been previously investigated longitudinally from embryonic to adult stages. As in the brain, we found that CD11c^+^ microglia were found in the spinal cord at E12.5 and E14.5 and confirmed that all cells were positive for IBA1 (Fig. 5A). Through the gating processes (Fig. 2A), CD11c^+^ microglia were also clearly detected in the spinal cord at P4 (Fig. 5B), and their percentage of total microglia was approximately 30%. As observed in the brain, the CD11c^+^ population at P4 highly expressed *Igf1*, *Clec7a*, and *Trem2* mRNAs (Fig. 5C). After P4, this percentage decreased sharply at P14 (Fig. 5D). Consistent with these data, immunohistochemical analysis showed that the proportion of CD11c^+^ microglia, which were abundant at P4, was markedly reduced at P14 (Fig. 6A). Interestingly, in stark contrast to the results of the forebrain, the proportion of CD11c^+^ microglia increased in the spinal cord at P21, and the percentage was increased by approximately 10% on average over P56 (Fig. 5D). The proportion of CD11c^high^ microglia increased from P14 to P21 (Fig. 5D). Notably, the localization of CD11c^+^ microglia in the spinal cord was greatly altered at P21 from gray matter (GM) at P4 and P7 to white matter (WM) at P21 and P56 (Fig. 6A, B). These results indicated the unique spatiotemporal dynamics of CD11c^+^ microglia in the spinal cord.

## Discussion

In this study, by comparatively analyzing six different CNS regions, we demonstrated the spatial and temporal characterization of CD11c^+^ microglia in these regions from pre/postnatal development to adult stages. Our data extend earlier findings obtained from the analysis of the whole brain and a specific brain region and time points [12, 13, 25], and provide evidence indicating that the dynamics of CD11c^+^ microglia differ between regions. Indeed, the proportion of CD11c^+^ microglia to total microglia varied among CNS regions; a higher proportion was observed in the olfactory bulb (P4) and cerebellum (P7), where approximately half of the total microglia were CD11c^+^. In the cerebellum, the temporal pattern of CD11c^+^ microglia (peak observed at P7) also differed from that of other regions. Furthermore, from P4/P7 onward, the proportion of CD11c^+^ microglia decreased in all regions, but the pattern was slightly different. It is unlikely that the observed proportional dynamics in CD11c^+^ microglia are due to a change in the promoter activity of *Itgax* alone. CD11c^+^ microglia found in the forebrain and spinal cord commonly had higher expression of *Igf1*, *Clec7a*, and *Trem2*, which have been shown to be among the characteristic genes expressed in CD11c^+^ microglia [3, 11, 12]. Therefore, the dynamics of CD11c^+^ microglia in the CNS from embryonic development to adult stages found in this study could reflect a shift between the CD11c^neg^ and CD11c^+^ population/state.

This study also longitudinally analyzed CD11c^+^ microglia in the developing and adult spinal cords and demonstrated their unique spatiotemporal dynamics for the first time. As in the brain, CD11c^+^ spinal microglia were found at E12.5, became abundant at around P4, and their proportion sharply decreased by P14. However, the CD11c^+^ (especially CD11c^high^) proportion increased at P21 and the increase gradually accelerated thereafter, a pattern that was not observed in the brain regions. More interestingly, during this period, the distribution of CD11c^+^ spinal microglia also markedly changed from the GM at P4/P7 to the WM from P21 onward. Given that the characteristic genes expressed in CD11c^+^ microglia were commonly found at P4 (Fig. 5C) and adult stage [21], the CD11c^+^ state of spinal microglia could spatially shift from the GM to the WM during the postnatal stage. In addition, the appearance of CD11c^+^ microglia in the WM may involve local self-expansion of these cells via proliferation. Alternatively, CD11c^+^ microglia may migrate from the GM to the WM during this period, as these cells have been reported to express high levels of chemokine receptors [12, 18, 21]. This could be determined by further investigations using tools that enable cell-fate mapping analysis (e.g., *Itgax-CreERT2* mice). These tools would also be useful to investigate their responsiveness to extracellular substances (e.g., ATP), function and role during CNS development, which are also important topics for future study.

However, the mechanisms underlying the spatiotemporal dynamics observed in the brain and the spinal cord require further investigation. Given that postnatal CD11c^+^ microglia are preferentially distributed in the WM and associated with myelinated fibers [8, 11, 12, 18, 23], CD11c^+^ microglia in this area may be related to the process of myelination/demyelination. Indeed, the emergence of CD11c^+^ microglia in various CNS disease models is accompanied by demyelination [20, 21, 26–29]. The appearance of CD11c^+^ microglia is attenuated by the loss of AXL [21], which are molecules that have been implicated in the phagocytosis of myelin debris [30]. Given the gene profile and function of CD11c^+^ microglia [3, 11, 12], their appearance may be linked to their role in spatiotemporally controlled myelination in the brain and spinal cord. CD11c^+^ microglia highly express *Igf1* [12, 21], a factor implicated in the myelination of the corpus callosum [12]. Conditional knockout of IGF1 in CD11c^+^ cells results in demyelination during early postnatal development [12]. Thus, it is conceivable that the appearance of CD11c^+^ microglia may be programmed by myelination/demyelination states in different contexts of normal development and disease. However, CD11c^+^ microglia are present in the forebrain and other regions during prenatal development (e.g., E12.5/14.5), a period much earlier than the development of myelin [31]. Thus, microglia in the brain and spinal cord acquire the CD11c^+^ state from the prenatal stage, but their regulation and role in CNS development are distinct between the pre- and postnatal phases. Consistently, a recent study conducting the comprehensive transcriptomic analysis of microglia during the early embryonic stage and their role in prenatal development demonstrated that microglia at E15 have a core gene signature characterized in previously reported postnatal ATM, including *Itgax*, *Spp1* and *Clec7a*, genes that are shared with CD11c^+^ microglia, and that prenatal ATM-like microglia have repair properties that are necessary for the maintenance of structural integrity during the development of the cerebral cortex [15].

In this study, we analyzed CD11b^+^/CD45^low^/CD206^neg^ cells as microglia, but some papers have reported that immature microglia in an early developmental stage express CD206 [4, 7]. A recent study has also shown that the proportion of CD206^+^ cells per total microglia in the mouse brain peaks at E13.5 (but it markedly decreases at E14.5 and later time points) [32]. Thus, a part of microglia at E12.5 may be excluded from the analysis of CD11c^+^ microglia at this time point, which was a limitation in our study.

In summary, our longitudinal comparative study of CD11c^+^ microglia from the early postnatal to adult CNS demonstrated that the spatial and temporal dynamics of this population vary between the brain and spinal cord regions. In humans, CD11c^+^ microglia-resembling population has recently been found in the brain during the developmental and adult stages, and disease conditions [4, 15, 33]. Our findings improve our understanding of the nature of microglial heterogeneity and its dynamics in the CNS.

## Methods

### Animals

Male/female *Itgax-Venus* mice (B6.Cg-Tg(*Itgax-Venus*)1Mnz/J) [34] were purchased from Jackson Laboratory (Bar Harbor, ME). Mice from E12.5 to P56 were used in each experiment. Mice were housed in groups at a temperature of 22 ± 1 °C with a 12-hour light–dark cycle, and were fed food and water ad libitum. All animal experiments were conducted according to relevant national and international guidelines contained in the ‘Act on Welfare and Management of Animals’ (Ministry of Environment of Japan) and ‘Regulation of Laboratory Animals’ (Kyushu University) and under the protocols approved by the Institutional Animal Care and Use committee review panels at Kyushu University.

### FACS

As previously described [21, 35], postnatal *Itgax-Venus* mice were deeply anesthetized by intraperitoneal (i.p.) injection of pentobarbital (P14, P21, P28, and P56) or hypothermia for 2–4 min until movement ceased (P0, P4, and P7), and perfused transcardially with phosphate-buffered saline (PBS) to remove circulating blood from the vasculature. The brain and spinal cord were rapidly and carefully removed and placed in ice-cold Hanks’ balanced salt solution (HBSS). The brain was further divided into five regions (forebrain, olfactory bulb, diencephalon/midbrain, cerebellum, and pons/medulla) as shown in Fig. 1A, and the choroid plexus was removed from the forebrain and cerebellar samples. To analyze CD11c^+^ microglia during prenatal development, whole brains were isolated from E12.5–E16.5 *Itgax-Venus* mice. The removed brain and spinal cord tissues were roughly minced and homogenized using a Potter tissue grinder in HBSS containing 15 mM HEPES buffer and 0.54% glucose. After spinning down, the cell suspension was separated by 37% Percoll (Sigma, St. Louis, MO) gradient centrifugation at 800 × g for 30 min at 4 °C with no brake. The pellet containing microglia at the bottom of the tube was then carefully collected and washed twice with PBS containing 2% fetal bovine serum (FBS) and 10 mM EDTA. Cells were treated with Fc Block (1:200; 553,142, BD Biosciences, San Jose, California) for 10 min at 4 °C before incubation with the primary antibodies [CD11b-BV786 (1:400; 740,861, BD Biosciences, San Jose, California), CD45-APC/Cyanine7 (1:400; 103,116; BioLegend, San Diego, California), and CD206-APC (1:200, 141,708; BioLegend, San Diego, California)] for 40 min at 4 °C. After washing, cells were analyzed and sorted using a CytoFLEX SRT (Beckman Coulter, Pasadena, California) and FlowJo software (TreeStar). Our criterion for CD11c^+^ microglia was CD11b^+^ CD45^low^ CD206^neg^ cells with Venus fluorescence with an intensity higher than the Venus intensity observed in CD11b^+^ CD45^low^ CD206^neg^ cells of wild-type mice as described previously [36, 37].

### Quantitative PCR (qPCR)

The sorted cells (5000 cells/sample) were subjected to total RNA extraction using the Quick-RNA Micro-Prep kit (ZYMO, Irvine, California). As described previously [21] the extracted RNA was transferred to reverse transcriptional reaction with Prime Script reverse transcriptase (Takara, Japan). Quantitative PCR (qPCR) was performed with FastStart Essential DNA Probes Master (Roche, Switzerland) using a QuantStudio 3 real-time PCR system (Thermo Fisher Scientific). Expression level of mRNA of each gene was normalized to the values for *Actb* mRNA. The sequences of TaqMan primer pairs and probe were described below: *Actb*: 5’-FAMCCTGGCCTCACTGTCCACCTTCCA-TAMRA-3’ (probe), 5’-CCTGAGCGCAAGTACTCTGTGT-3’ (forward primer), 5’-CTGCTTGCTGATCCACATCTG-3’ (reverse primer); *P2ry12*: 5’-/56-FAM/CCATGGATG/ZEN/TGCCTGGTGTCAACA/3IABkFQ/-3’ (probe), 5’-CCAGTCTGCAAGTTCCACTAAC-3’ (forward primer), 5’-GAGAAGGTGGTATTGGCTGAG-3’ (reverse primer); *Igf1*:5’-/56-FAM/TCCGGAAGC/ZEN/AACACTCACATCCACAA/3IABkFQ/-3’ (probe), 5’-AGTACATCTCCAGTCTCCTCA-3’ (forward primer), 5’-ATGCTCTTCAGTTCGTGTGT-3’ (reverse primer); *Clec7a*: 5’-/56-FAM/TCTTCACCT/ZEN/TGGAGGCCCATTGC/3IABkFQ/-3’(probe), 5’-TTCAGCACTCAAGACATCCAT-3’ (forward primer), 5’-CCACTACTACCACAAAGCACA-3’ (reverse primer); *Trem2*:5’-/56-FAM/TCCCAAGCC/ZEN/CTCAACACCACG /3IABkFQ/-3’ (probe),5’-GACCTCTCCAGTTTCTC-3’ (forward primer), 5’-GCTTCAAGGCGTCATAAGTACA-3’ (reverse primer).

### Immunohistochemistry

According to methods of our previous papers [21, 38], postnatal mice were deeply anesthetized by pentobarbital and perfused transcardially with PBS, followed by ice-cold 4% paraformaldehyde (PFA)/PBS. Postnatal brain and spinal cord were fixed for 4–6 h and 3–4 h, respectively, in 4% PFA at 4 ℃. Embryonic brains and spinal cord were isolated from E14.5 mice without transcardial PBS perfusion and were immersion fixed for 6 h in 4% PFA at 4 ℃. Embryo and postnatal tissues were placed in 30% sucrose for 48 h at 4 °C and embedded in Tissue-Tek OCT compound (Sakura Finetek, Japan). Cryosections were cut at a thickness of 20 μm, blocked with PBS containing 5% bovine serum albumin and 1% normal donkey serum, and then permeabilized with 0.5% Triton X-100 in blocking solution. The primary antibodies IBA1 (1:1000; 234 004, Synaptic systems, Goettingen, German) and GFP (1:1000; 598, MBL Life science, Tokyo, Japan) were added for 48 h at 4 °C. Tissue sections were incubated with secondary antibodies conjugated to Alexa Fluor 488 (1:1000; A-21,206, Thermo Fisher Scientific, Waltham, MA) and 546 (1:1000; 706-165-148, Jackson immunoReseach LABORATORIES INC., West Grove, Pennsylvania) and mounted with ProLong Glass Antifade Mountant (Thermo Fisher Scientific, Waltham, MA). Tissue sections were analyzed using LSM700 Imaging System (ZEN 2012, Carl Zeiss).

### Statistical analysis and data visualization

All data were shown as means ± SEM. Statistical significance was determined using the unpaired t-test or one-way ANOVA with post hoc Tukey’s multiple comparison test using GraphPad Prism 7 software. Differences were considered significant at *P* < 0.05.

## Data Availability

Requests for materials and correspondence should be addressed to M.T. (tsuda@phar.kyushu-u.ac.jp).

## Abbreviations

CNS: Central nervous system
E12.5: Embryonic day 12.5
FACS: Fluorescence-activated cell sorting
GM: Gray matter
HBSS: Hanks’ balanced salt solution, IBA1: ionized calcium-binding adaptor protein 1
P0: Postnatal day 0
PBS: Phosphate-buffered saline
PFA: Paraformaldehyde
qPCR: Quantitative polymerase chain reaction
WM: White matter
